# The characteristic expression of circulating *MicroRNAs* in osteoporosis: a systematic review and meta-analysis

**DOI:** 10.3389/fendo.2024.1481649

**Published:** 2024-12-16

**Authors:** Jie Gao, Xiuzhen Zhang, Jing Ding, Houli Zhang, Xu Zhang, Juan Jiang, Wenwen Chen

**Affiliations:** ^1^ Department of Pharmacy, the Second Affiliated Hospital of Shandong First Medical University, Tai’an, China; ^2^ Department of Stomatology, the Second Affiliated Hospital of Shandong First Medical University, Tai’an, China

**Keywords:** microRNA, expression, diagnosis, osteoporosis, meta-analysis

## Abstract

**Objective:**

To evaluate the characteristics of the circulating microRNA expression profiles in patients with osteoporosis.

**Methods:**

A systematic literature search was performed using the Web of Science, PubMed, Embase, Cochrane Library, China National Knowledge Infrastructure (CNKI), VIP, and WANFANG databases from inception until 1 March 2024. The search strategy employed keywords, encompassing “osteoporosis”, “bone loss”, or “osteopenia” and “miRNA” or “microRNA”. The Newcastle-Ottawa Scale (NOS) quality assessment scale was used to evaluate the methodological quality. Heterogeneity tests and statistical analyses of all data were performed by Stata 16.0. The differences in microRNA levels between groups were illustrated by the weighted mean difference (WMD) and 95% confidence interval (95% CI).

**Results:**

A total of 27 studies were included and analyzed in the meta-analysis, with 2,263 participants. The results showed that *miR-21-5p* (WMD 0.88, 95% CI: 0.22 to 1.55), *miR-125b-5p* (WMD 6.63, 95% CI: 0.19 to 13.08), *miR-483-5p*(WMD 6.43, 95% CI: 3.26 to 9.61), *miR-133a* (WMD 1.43, 95% CI: 1.39 to 1.47), *miR-422a* (WMD 1, 95% CI: 0.28 to 1.72), and *miR-214-3p* (WMD 2.03, 95% CI: 0.14 to 3.92) were significantly upregulated, and *miR-497-5p* (WMD -0.57, 95% CI: -0.98 to -0.17) was significantly downregulated.

**Conclusion:**

*miR-21-5p*, *miR-125b-5p*, *miR-483-5p*, *miR-133a*, *miR-497-5p*, *miR-422a*, and *miR-214-3p* might serve as potential diagnostic biomarkers for osteoporosis. In the future, integrating these miRNAs to build a diagnostic model might be a promising diagnosis strategy for osteoporosis.

**Systematic review registration:**

https://www.crd.york.ac.uk/PROSPERO/ **, identifier CRD42023481209.**

## 1 Introduction

Osteoporosis (OP), a progressive skeletal disorder characterized by reduced bone mineral density, compromised bone strength, and increased risk of fracture, has garnered widespread attention because it has a significant impact on health-related quality of life (1). Nearly 200 million people worldwide are diagnosed with osteoporosis each year, and nearly 9 million osteoporotic fractures occur each year (2). Hip and vertebrae fractures are associated with particularly high morbidity and mortality, posing a high socioeconomic burden on overstretched health systems (3). Therefore, fracture prevention through early diagnosis is the primary goal of osteoporosis management. However, the onset of osteoporosis is usually asymptomatic, which highlights the need for a strategy that allows a quick and effective diagnosis of osteoporosis to mitigate further disease progress and subsequent fractures (4).

Currently, bone mineral density (BMD), measured using dual-energy x-ray absorptiometry (DXA), is employed to diagnose osteoporosis (5). Higher BMD indicates denser, stronger bones and lower BMD indicates less dense, weaker bones. Notably, not only bone mass but also bone quality is a factor in osteoporosis. Unfortunately, DXA cannot accurately assess bone quality (6). In addition, spinal deformities, previous compression fractures, and aortic atherosclerosis can lead to an increase in effective x-ray uptake, resulting in a pseudo-elevation of the T-Score (7, 8). Overall, DXA scans are not accurate enough to diagnose osteoporosis. Therefore, an effective diagnostic indicator to identify osteoporosis is urgently required.

*MicroRNAs* (miRNAs), which are non-encoded, endogenous, and single-stranded RNAs of ~22 nucleotides, are important regulators of numerous biological processes through the posttranscriptional regulation of gene expression (9, 10). A large number of *in vitro* and *in vivo* studies suggest that miRNAs are involved in cell differentiation, proliferation, autophagy, and apoptosis in the bone microenvironment. Differential expression of miRNAs in different stages of the development of osteoporosis have shown that they are closely related to the occurrence and development of osteoporosis (11).

Recently, numerous research studies have focused on circulating miRNAs as a potential biomarker for the early detection of osteoporosis (12). Some studies indicated up or downregulation of diverse miRNAs in osteoporotic postmenopausal women compared with healthy postmenopausal women (13). Due to different technological platforms and small sample sizes among various studies, conflicting results regarding the direction of regulation have been found for some miRNAs. Furthermore, the characteristic expression of circulating miRNAs in osteoporosis has not been accurately evaluated. This meta-analysis was performed to further clarify the characteristics of the circulating miRNA expression profiles in osteoporosis, and explore the potential value of circulating miRNAs for the diagnosis of osteoporosis.

## 2 Methods

This systematic review and meta-analysis was conducted in line with the Preferred Reporting Items for Systematic Reviews and Meta-Analyses (PRISMA) statement. The protocol for this meta-analysis was prospectively registered in the PROSPERO database (CRD42023481209).

### 2.1 Data sources and retrieval strategy

A systematic literature search was performed using the Web of Science, PubMed, Embase, Cochrane Library, CNKI, VIP, and WANFANG databases. The literature search included studies published in English or Chinese from inception until 1 March 2024. The search strategy employed the following keywords: “osteoporosis”, “bone loss”, or “osteopenia” and “miRNA” or “microRNA”. For the identification of additional relevant studies, the reference lists of related reviews and included articles were screened manually.

### 2.2 Selection criteria

The inclusion criteria were defined as (1): the study should have the miRNA expression profiles of patients with osteoporosis (2); osteoporosis patients were included in the experimental group, and healthy individuals were included in the control group (3); the relative miRNA expression must be profiled by RT-qPCR (4); the sample size was reported (5); the mean and standard deviation of the miRNA expression profiles could be obtained; and (6) all the patients with osteoporosis were diagnosed by DXA. The exclusion criteria were as follows (1): studies on animals (2); simple descriptive literature without a control group (3); review literature, case report, abstract, and letter; and (4) literature for which the relevant data could not be obtained.

### 2.3 Data extraction

The following study data was extracted: first author, year of publication, country, age, sex, source of samples, miRNA expression profile, assay type, and sample size. Two investigators independently extracted the data following the pre-defined inclusion and exclusion criteria. If different sample sources were provided in the same study, data extraction and analysis were carried out respectively. Any discrepancies were resolved through discussions and, if necessary, a third senior investigator was consulted until a consensus was reached. The corresponding authors of the original articles were contacted to obtain relevant data that were not present in the full text and supplementary information. If data were only shown by graphs, GetData Graph Digitizer software (version 2.26) was used to extract numerical values.

### 2.4 Quality assessment

Two independent investigators used the Newcastle-Ottawa Scale (NOS) to assess the quality of the included studies. The scale involved three aspects with a total of 9 points: the selection of cohorts (0–4 points), the comparability of cohorts (0–2 points), and the assessment of the outcome (0–3 points). Quality was classified as high with an NOS score of ≥6 points and low with an NOS score of <6 points (14). Disagreements were resolved until a consensus was reached by mutual discussion with a third senior investigator.

### 2.5 Statistical analysis

Stata software (version 16.0, Stata Corp LP, College Station, TX, USA) was used to analyze the data. The weighted mean difference (WMD) and 95% confidence interval (95% CI) were calculated to indicate the effect size of the differences in miRNA expression levels between groups. The heterogeneity of the included studies was usually tested by Cochrane’s Q test and I^2^ statistic. For Cochrane’s Q test, P < 0.1 indicated significant heterogeneity between the studies. The fixed effect model was selected if I^2^ was < 50% and the random effect model was selected if I^2^ was ≥ 50%. Subgroup and regression analyses were conducted to explore the possible sources of heterogeneity. A sensitivity analysis was performed to analyze the stability and reliability of the effect size using the “remove one study” method. Finally, Egger’s and Begg’s tests, as well as the visual inspection of funnel plots, were used to assess any potential publication bias.

## 3 Results

### 3.1 Search results and study characteristics

**Figure 1:**
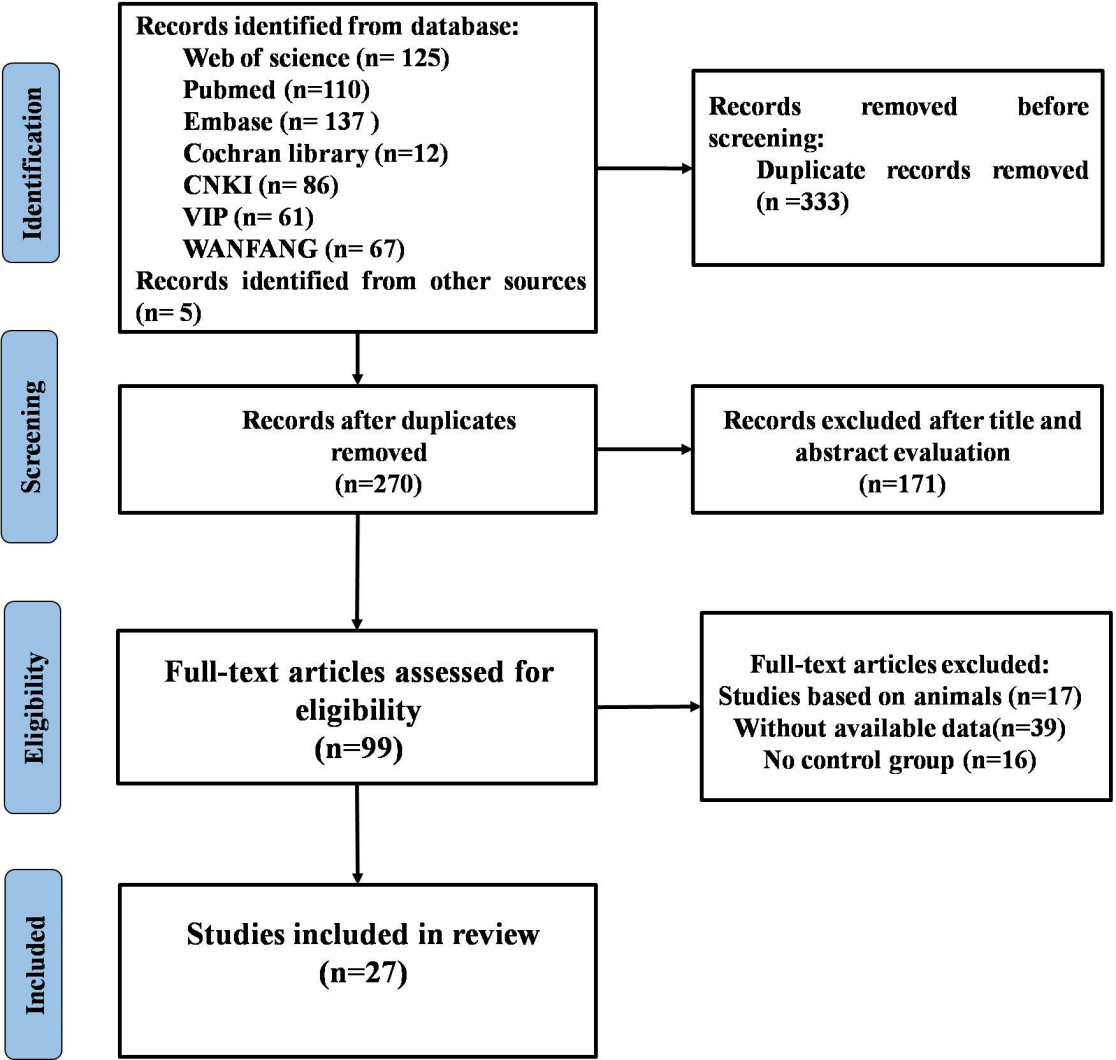
The flow diagram for study selection.

The selection process of the studies is presented in **Figure 1**. Our search returned a total of 603 studies. After the elimination of duplicates, 270 articles were reviewed. Of these, 171 were excluded by browsing the title and abstract, leaving 99 full-text studies to be reviewed. Finally, 27 studies that met the inclusion criteria were included and analyzed in the meta-analysis, with 2,263 participants. Details of the included studies are shown in **Supplementary Table 1**. All the studies were published between 2014 and 2023, with 19 conducted in Asia, 4 in Europe, 3 in North America, and 1 in Africa. The average age of all participants ranged from 39.1 to 80 years old, and most of the participants were female.

### 3.2 Quality evaluation results

The quality of the included studies was evaluated by applying the NOS, as shown in **Table 1**. The NOS scores ranged from 7 to 8, and, as such, all the included studies were determined to be of high quality.

**Table 1:** Quality assessment of the included studies.

Studies	Selection	Comparability	Exposure	Scores
Wang 2023	***	**	**	7
Al-Rawaf 2021	****	**	**	8
Gu 2020	***	**	**	7
Cao 2014	****	*	**	7
Alrashed 2022	***	**	**	7
Li 2018	***	**	**	7
Li 2020	***	**	**	7
Li 2014	***	**	**	7
Suarjana 2019	****	**	**	8
Mohammadisima 2023	****	**	**	8
Chen 2017	****	*	**	7
Bedene 2016	****	**	**	8
Wang 2021	***	**	**	7
Mandourah 2018	***	**	**	7
Ma 2020	***	**	**	7
Nesma2019	***	**	**	7
Wang 2020	***	**	**	7
Seeliger 2014	****	*	**	7
Wang 2012	****	*	**	7
Al-Rawaf 2023	****	**	**	8
Ciuffi 2022	****	**	**	8
Xu 2021	***	**	**	7
Cong 2020	***	**	**	7
Chen 2019	****	**	**	8
Yang 2013	****	**	**	8
Zhao 2021	***	**	**	7
Wang 2018	***	**	**	7

* scores 1 point; ** scores 2 point; *** scores 3 point; **** scores 4 point.

### 3.3 Results of the meta-analysis of the expression of each circulating miRNA in patients with osteoporosis

**Figure 2:**
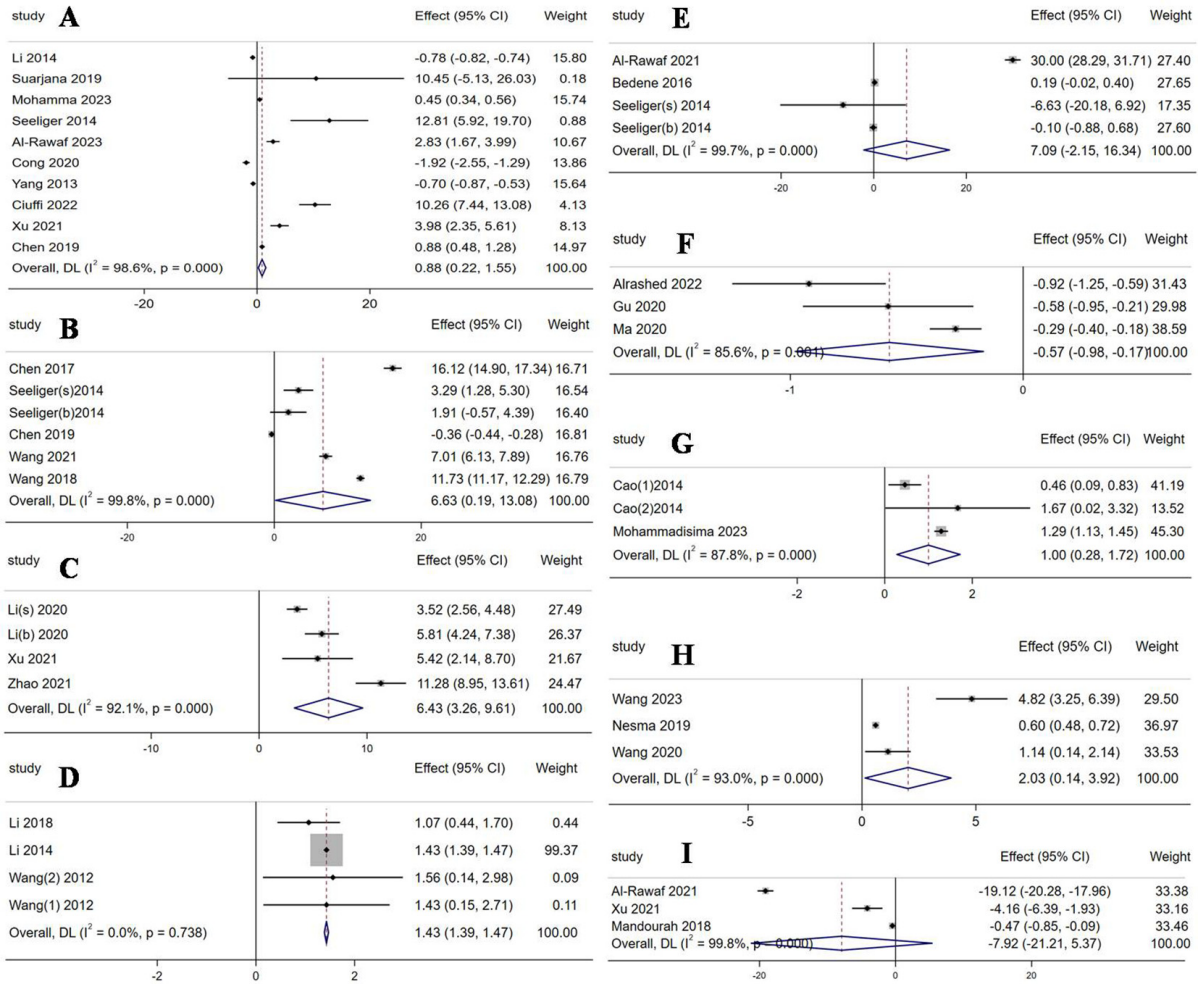
Forest plot of the expression of each circulating miRNA. **(A)** *miR-21-5p*; **(B)** *miR-125b-5p*; **(C)** *miR-483-5p*; **(D)** *miR-133a*; **(E)** *miR-148a-3p*; **(F)** *miR-497-5p*; **(G)** *miR-422a*; **(H)** *miR-214-3p*; **(I)** *miR-122-5p*.

Six miRNAs were evidently upregulated and had been screened out as follows: *miR-21-5p* (WMD 0.88, 95% CI: 0.22 to 1.55, p = 0.009, I^2^ = 98.6%, **Figure 2A**), *miR-125b-5p* (WMD 6.63, 95% CI: 0.19 to 13.08, p = 0.044, I^2^ = 99.8%, **Figure 2B**), *miR-483-5p* (WMD 6.43, 95% CI: 3.26 to 9.61, p < 0.001, I^2^ = 92.1%, **Figure 2C**), *miR-133a* (WMD 1.43, 95% CI: 1.39 to 1.47, p < 0.001, I^2^ = 0%, **Figure 2D**), *miR-422a* (WMD 1, 95% CI: 0.28 to 1.72, p = 0.007, I^2^ =87.8%, **Figure 2G**), and *miR-214-3p* (WMD 2.03, 95% CI: 0.14 to 3.92, p *=* 0.036, I^2^ =93%, **Figure 2H**). Only one miRNA, *miR-497-5p*, was downregulated with a mean difference of -0.57 (WMD -0.57, 95% CI: -0.98 to -0.17, p = 0.005, I^2^ =85.6%, **Figure 2F**). The other two miRNAs, *miR-148a-3p* (WMD 7.09, 95% CI: -2.15 to 16.34, p = 0.133, I^2^ =99.7%, **Figure 2E**) and *miR-122-5p* (WMD -7.92, 95% CI: -21.21 to 5.37, p = 0.243, I^2^ =99.8%, **Figure 2I**), were observed to not have significant differences in expression.

### 3.4 Subgroup analysis and meta-regression analysis

In order to explore the source of heterogeneity, a subgroup analysis should be carried out for *miR-21-5p*, *miR-125b-5p*, *miR-483-5p*, *miR-148a-3p*, *miR-497-5p*, *miR-422a*, *miR-214-3p*, and *miR-122-5p*. Due to a lack of publications, only *miR-21-5p* was analyzed based on the sample sources (**Supplementary Figure 1**). The result showed that *miR-21-5p* was significantly upregulated in serum samples (WMD 5.66, 95% CI: 2.37 to 8.96, p = 0.001, I^2^ = 93.5%), but was observed to have no significant expression differences in plasma samples (WMD 0.03, 95% CI: -0.92 to 0.98, p = 0.948, I^2^ = 99.4%). The result of the subgroup analysis, as well as a subsequent meta-regression analysis (p = 0.058), indicated that the sample source was not a potential source of heterogeneity for *miR-21-5p*.

### 3.5 Sensitivity analysis and publication bias test

After excluding any individual study, a sensitivity analysis revealed that the results for the miRNAs in the present study were stable (**Supplementary Figure 2**). All the studies included in the present meta‐analysis were symmetrically distributed in a funnel plot (**Supplementary Figure 3**). The p-values of Begg’s and Egger’s tests are shown in **Supplementary Table 2** (p > 0.05 for all), which indicated the absence of significant publication bias in the included studies.

## 4 Discussion

In the present meta-analysis, nine miRNAs were differentially expressed in more than one study for OP. Among these miRNAs, *miR-21-5p*, *miR-125b-5p*, *miR-483-5p*, *miR-133a*, *miR-422a*, and *miR-214-3p* were significantly upregulated, and *miR-497-5p* was significantly downregulated.

The dysregulation of *miR-21-5p* in OP has received a lot of attention from researchers in recent years. Notably, the direction of regulation for *miR-21-5p* was contradictory in the studies included in the meta-analysis, with seven studies reporting upregulation (15–21) and three studies reporting downregulation (22–24). This could be due to *miR-21-5p* being closely associated with both osteogenic and osteoclastic differentiation through a variety of mechanisms. *miR-21-5p* has been reported to promote osteogenesis by targeting the SOX2 (25), PLAP-1 (26), ACVR2B (27), ERK-MAPK (24, 28), Smad7-Smad1/5/8-Runx2 (29–31), PI3K/β-catenin (32), and PTEN/PI3K/Akt/HIF-1α pathways (33). The regulatory effects of *miR-21-5p* on osteoclasts are complex and involve multiple mechanisms. Sugatani et al. proposed a new molecular mechanism for osteoclastogenesis, namely the C-Fos/*miR-21*/PDCD4 positive feedback loop. C-Fos upregulated *miR-21* expression and inhibited PDCD4 expression, which in turn promoted osteoclastogenesis (34). Subsequent studies also confirmed that *miR-21* directly regulates osteoclast function by targeting PDCD4 (35–37). In addition, PTEN (38, 39), SKP2 (40), OPG (41), and FasL (42) have also been shown to be effective targets of *miR-21-5p* in promoting osteoclast differentiation. However, an inhibitory effect of *miR-21-5p* on osteoclast differentiation has also been reported, for instance, Huang et al. found that *miR-21-5p* was significantly decreased during osteoclast differentiation and that *miR-21-5p* inhibited osteoclast differentiation by acting on its target gene *SKP2* (40). In juvenile idiopathic arthritis, *miR-21-5p* could inhibit the production of osteoclasts from rheumatoid arthritis fibroblast-like synovial cells induced by M-CSF (43). Unsatisfactorily, in the aforementioned studies, *miR-21-5p* promoted both osteogenic and osteoclastic differentiation, which is contradictory. This prevented us from accurately describing the mechanistic role of *miR-21-5p* in the pathogenesis of osteoporosis, so further studies are needed. Nevertheless, the results of this meta-analysis indicated that the level of *miR-21-5p* was upregulated in patients with osteoporosis, which was consistent with the result of a previously reported meta-analysis based on the robust rank aggregation method (13). Based on this finding, it could be considered that the expression level of *miR-21-5p* is related to the occurrence of osteoporosis, suggesting that *miR-21-5p* might be a promising biomarker for the diagnosis of osteoporosis. However, it should be noted that *miR-21-5p* was also associated with cardiovascular diseases (44), cancer (45), and other bone diseases (46), so a differential diagnosis is required in clinical practice.

For the expression level of *miR-125b-5p* in osteoporosis patients, four included studies reported upregulation (17, 47–49) and only one study reported downregulation (21). Wang et al. discovered that overexpression of *miR-125b-5p* was responsible for the development of postmenopausal osteoporosis and promoted its progression through the *TRAF6* gene via the JAK2/STAT3 pathway (47). Xue et al. found that *miR-125b* attenuated the osteoblastic differentiation of periodontal ligament cells by targeting NKIRAS2 and enhancing NF-κB signaling (50). Wang et al. demonstrated that *miR-125b-5p* regulated the osteogenic differentiation of human mesenchymal stem cells by targeting BMPR1b and that inhibiting *miR-125b-5p* expression could enhance the capacity of bone defect repair *in vivo (* 51). Huang et al. observed that the overexpression of *miR-125b-5p* inhibited osteoblastic differentiation by directly targeting Cbfβ and indirectly acting on Runx2 at the early stage of osteoblastic differentiation (52). On the contrary, a study from Japan showed that *miR-125b-5p* inhibited osteoclast formation by targeting Prdm1, encoding a transcriptional repressor of anti-osteoclastogenesis factors (53). Chen et al. found that irisin can upregulate the expression level of *miR-125b-5p* by targeting SIPA1L2, which regulated the Rap1/PI3K/AKT axis and finally increased the expression levels of the chondrogenic differentiation genes *COL2A1*, *ACAN*, and *SOX9 (* 54). Overall, the available fundamental research showed that *miR-125b-5p* was more inclined to inhibit osteogenesis differentiation. The level of *miR-125b-5p* was consistently upregulated in patients with osteoporosis in the present meta-analysis. This suggests that *miR-125b-5p* might be a potential biomarker for the diagnosis of osteoporosis. However, it should be noted that *miR-125b-5p* has also been associated with stroke (55), Alzheimer’s disease (56), and cancer (57), so a differential diagnosis is required.

In the present study, *miR-483-5p* was evaluated in three studies (20, 58, 59), and all the studies indicated it was upregulated in osteoporosis. Li et al. revealed that *miR-483-5p* is involved in the pathogenesis of osteoporosis by reducing the apoptosis of osteoclasts (58). Zhao et al. also indicated that *miR-483-5p* could inhibit osteogenic differentiation by inhibiting SATB2 and activating the PI3K/AKT pathway (59). Peng et al. demonstrated that *miR-483-5p* regulated the RAS/MEK/ERK signaling pathway by targeting RPL31 and inhibiting its expression, thereby playing an inhibitory role in osteogenic differentiation (60). In light of these findings, *miR-483-5p* could be used as a diagnostic marker for osteoporosis. However, a differential diagnosis is necessary based on the association of *miR-483-5p* with other diseases (61, 62).

*miR-133a* was overexpressed in all the included studies (22, 63, 64). Wang et al. revealed that the overexpression of *miR-133a* suppressed osteoblast differentiation of bone marrow mesenchymal stem cells and silencing *miR-133a* resulted in positive effects on glucocorticoid-treated mesenchymal stem cells and on bone loss in glucocorticoid-induced osteoporosis animal models through the MAPK/ERK signaling pathway by targeting FGFR1 (65, 66). Li et al. found that *miR-133a* knockdown altered the levels of osteoclastogenesis-related factors in serum, increased lumbar spine BMD, and changed bone histomorphology in ovariectomized rats (63). In contrast, Zhou et al. suggested that *miR-133a* in osteoblasts significantly alleviated bone loss and microstructural and biomechanical properties in mice with mechanical unloading, contributing to osteopenia alleviation. Furthermore, *miR-133a* could also restrain osteoclastogenesis (67). However, in the present article, the direction of regulation for *miR-133a* in different studies was consistent, which meant that *miR-133a* might be a promising biomarker for osteoporosis diagnosis. Of course, a differential diagnosis is also required (68, 69).

Based on three studies, *miR-497-5p* was downregulated in osteoporotic patients with agreement on the direction of change (70–72). Zhao et al. showed that *miR-497-5p* enhanced osteogenic differentiation by repressing HMGA2 and impairing the JNK signaling pathway based on the MC3T3-E1 cell line (73). Lu et al. discovered the downregulation of SNHG1 and HIF1AN, in contrast with an elevation in *miR-497-5p* levels throughout osteogenic differentiation. By influencing *miR-497-5p*, SNHG1 exhibited the ability to modulate HIF1AN, thereby inhibiting osteogenic differentiation (74). Gu et al. suggested that *miR-497-5p* upregulation promoted osteoblast viability and collagen synthesis by activating the TGF-β1/Smads signaling pathway (70). Taken together, the discoveries of the mechanism studies were consistent with the result of the present meta-analysis, which suggested that *miR-497-5p* might be a novel reference for the diagnosis of osteoporosis. Notably, like other miRNAs, *miR-497-5p* was also involved in the development of other diseases (75–77).

For the expression direction of *miR-422a*, all the studies reported upregulation (16, 78). Baloun et al. observed that the serum concentration of *miR-422a* was positively correlated with markers of bone remodeling (β-CTX and P1NP), suggesting a role in the pathogenesis of osteoporosis (79). Cao et al. found significant upregulation of *miR-422a* in the low BMD group compared with the high BMD group using RT-qPCR analysis (P = 0.029). Furthermore, through bioinformatic target gene and RT-qPCR analyses, Cao et al. identified several potential target genes (*CBL*, *CD226*, *IGF1*, *PAG1*, and *TOB2*) of *miR-422a* that inhibit osteoclastogenesis and the expression of these genes correlated negatively with *miR-422a* expression (78). In our meta-analysis, the expression level of *miR-422a* was upregulated in osteoporosis patients. However, there are insufficient studies on the specific mechanism of *miR-422a* in the pathogenesis of osteoporosis. Therefore, further basic and clinical studies were required to confirm whether *miR-422a* could be a potential biomarker for osteoporosis diagnosis.

Consistent results for the direction of regulation were also found for *miR-214-3p*, and its expression level was elevated in patients with osteoporosis (80–82). *miR-214-3p* had been reported to suppress osteogenic differentiation by targeting Osterix (83), ATF4 (84, 85), and FGFR1 (86), and promote osteoclastogenesis by targeting phosphatase and tensin homolog (PTEN) (87). Furthermore, downregulation of *miR-214-3p* by Circ-ITCH (88), PTENP1 (82), and Nrf2 (89) has been reported to promote osteogenic differentiation and inhibit osteoclast differentiation, attenuating osteoporosis. These discoveries revealed that *miR-214-3p* has a crucial role in osteoporosis and might be a promising biomarker for the diagnosis of osteoporosis. Notably, a differential diagnosis is necessary due to the association of *miR-214-3p* with other diseases (90, 91).

In this meta-analysis, no significant expression differences were found for *miR-122-5p* (20, 92, 93) and *miR-148a-3p* (17, 92, 94) between patients with osteoporosis and healthy individuals. Only two studies reported an inhibitory effect of *miR-122-5p* on osteoblast proliferation/differentiation in osteoporosis (95, 96). Although *miR-148a-3p* has been reported to prevent osteoblast differentiation and bone remodeling in several fundamental studies (97–100), its expression direction in clinical studies was contradictory (17, 92, 94). Therefore, further studies are still required to confirm whether these two miRNAs can be used as diagnostic biomarkers for osteoporosis.

There were several limitations in our meta-analysis. First, although we had comprehensively searched enough databases, the number of studies and sample sizes of the studies available for meta-analysis were still small due to miRNA not being routinely used for the diagnosis of osteoporosis in clinical practice, which might impact the statistical power and generalizability of the results. Studies with larger sample sizes and deeper data analyses are needed to validate our findings. Second, data was partially obtained from bar charts or scatter charts that might not be accurate. Third, there was evident heterogeneity, reflected in the wide variation in miRNA expression in the osteoporosis population, limiting their use as diagnostic biomarkers for osteoporosis.

In conclusion, even with the limitations of the meta-analysis, it can be argued that *miR-21-5p*, *miR-125b-5p*, *miR-483-5p*, *miR-133a*, *miR-148a-3p*, *miR-497-5p*, *miR-422a*, *miR-214-3p*, and *miR-122-5p* are associated with osteoporosis and could be potential diagnostic biomarkers for osteoporosis. In the future, well-designed exhaustive studies should be conducted to validate the diagnostic value of these miRNAs for osteoporosis.

## Data Availability

The original contributions presented in the study are included in the article/**Supplementary Material**. Further inquiries can be directed to the corresponding authors.
