# Supplementary material for: The characteristic expression of circulating MicroRNAs in osteoporosis: a systematic review and meta-analysis
Source: Front Endocrinol (Lausanne). 2024 Dec 16;15:1481649. doi: 10.3389/fendo.2024.1481649 (PMC11682891; doi:10.3389/fendo.2024.1481649)
Supplement: Supplementary file 1 [file DataSheet1.zip › supplementary files/Supplemental Table 1. Characteristics of included studies.docx]

Supplemental Table 1. Characteristics of included studies.

| First author | Year | Country | Mean age range | Female  (%) | Source | MiRNA expression profile | Assay type | Sample size | |
| --- | --- | --- | --- | --- | --- | --- | --- | --- | --- |
|  |  |  |  |  |  |  |  | Cases | Controls |
| Wang ^[80]^ | 2023 | China | 65-75 | UN | bone | miR-214 (up) | RT‒qPCR | 6 | 6 |
| Al-Rawaf ^[98]^ | 2021 | Saudi Arabia | 62.6-63.9 | 100 | serum | miR-148a(up) | RT‒qPCR | 55 | 45 |
|  |  |  | 62.6-63.9 | 100 | serum | miR-122-5p(down) | RT‒qPCR | 55 | 45 |
| Gu ^[70]^ | 2020 | China | UN | 22.5 | bone | miR-497(down) | RT‒qPCR | 20 | 20 |
| Cao(2) ^[78]^ | 2014 | America | 57-68 | 100 | plasma MonocyMonocyte | miR-422a(up) | Array-Analyses | 10 | 10 |
| Cao(1) ^[78]^ |  |  | 57-68 | 100 | plasma | miR-422a(up) | RT‒qPCR | 10 | 10 |
| Alrashed ^[71]^ | 2022 | Saudi Arabia | 58.77-59.36 | 70 | bone | miR-497-5p(down) | RT‒qPCR | 25 | 25 |
| Li ^[63]^ | 2018 | China | 59-80 | 100 | serum | miR-133a(up) | RT‒qPCR | 10 | 10 |
| Li(s) ^[58]^ | 2020 | China | 59-62 | 100 | serum | miR-483–5p(up) | RT‒qPCR | 36 | 36 |
| Li(b) ^[58]^ |  |  | 59-62 | 100 | bone | miR-483–5p(up) | RT‒qPCR | 36 | 36 |
| Li ^[22]^ | 2014 | China | 56.5-57.5 | 100 | plasma | miR-21-5p(down) | RT‒qPCR | 40 | 40 |
|  |  |  | 56.5-57.5 | 100 | plasma | miR-133a(up) | RT‒qPCR | 40 | 40 |
| Suarjana ^[18]^ | 2019 | Indonesia | 58.5-62 | 100 | serum | miR-21-5p(up) | RT‒qPCR | 60 | 60 |
| Mohammadisima ^[16]^ | 2023 | Iran | 55.7-58.2 | 100 | plasma | miR-21-5p(up) | RT‒qPCR | 65 | 61 |
|  |  |  | 55.7-58.2 | 100 | plasma | miR-422a(up) | RT‒qPCR | 65 | 61 |
| Chen ^[49]^ | 2017 | China | 59-80 | 100 | serum | miR-125b-5p(up) | RT‒qPCR | 30 | 30 |
| Bedene ^[99]^ | 2016 | Slovenia | 61-62 | 100 | plasma | miR-148a-3p(up) | RT‒qPCR | 17 | 57 |
| Wang ^[47]^ | 2021 | China | UN | 100 | serum | miR-125b-5p(up) | RT-qPCR | 10 | 8 |
| Mandourah ^[93]^ | 2018 | United Kingdom | 67-70 | 85.71 | serum | miR-122-5p(down) | RT-qPCR | 51 | 12 |
|  |  |  |  |  |  |  |  |  |  |
| Supplemental Table 1. Characteristics of included studies (continued) | | | | | | | | | |
| Ma ^[72]^ | 2020 | China | 39.1-70.3 | 100 | serum | miR-497-5p(down) | RT-qPCR | 17 | 14 |
| Mohamad ^[81]^ | 2019 | Egypt | 53.64-59.54 | 60 | bone | miR-214(up) | RT-qPCR | 26 | 14 |
| Wang ^[82]^ | 2020 | China | UN | 100 | bone | miR-214(Up) | RT-qPCR | 30 | 15 |
| Seeliger(s) ^[17]^ | 2014 | Germany | 76.6-78.3 | 100 | serum | miR-21-5p(up) | RT‒qPCR | 30 | 30 |
| Seeliger(s) ^[17]^ |  |  | 76.6-78.3 | 100 | serum | miR-125b-5p(up) | RT‒qPCR | 30 | 30 |
| Seeliger(s) ^[17]^ |  |  | 76.6-78.3 | 100 | serum | miR-148a (up) | RT‒qPCR | 30 | 30 |
| Seeliger(b) ^[17]^ |  |  | 76.6-78.3 | 72.5 | bone | miR-125b-5p(up) | RT‒qPCR | 20 | 20 |
| Seeliger(b) ^[17]^ |  |  | 76.6-78.3 | 72.5 | bone | miR-148a (up) | RT‒qPCR | 20 | 20 |
| Wang(2) ^[64]^ | 2012 | America | 61.6-63.6 | 100 | plasma | miR-133a(up) | Array-Analysis | 10 | 10 |
| Wang(1) ^[64]^ |  |  | 61.6-63.6 | 100 | plasma | miR-133a(up) | RT‒qPCR | 10 | 10 |
| Al-Rawaf ^[15]^ | 2023 | Saudi Arabia | 43.4-43.5 | 100 | serum | miR-21-5p(up) | RT‒qPCR | 30 | 25 |
| Ciuffi ^[19]^ | 2022 | Italy | 66.8-67.1 | 19.14 | serum | miR-21-5p(up) | RT‒qPCR | 54 | 46 |
| Xu ^[20]^ | 2021 | China | 65-66.7 | 50 | plasma | miR-483-5p (up) | RT‒qPCR | 8 | 8 |
|  |  |  | 65-66.7 | 50 | plasma | miR-21-5p(up) | RT‒qPCR | 8 | 8 |
|  |  |  | 65-66.7 | 50 | plasma | miR -122-5p(down) | RT‒qPCR | 8 | 8 |
| Cong ^[23]^ | 2020 | China | 54.1-54.3 | 36.36 | plasma | miR-21-5p(down) | RT‒qPCR | 66 | 66 |
| Chen ^[21]^ | 2019 | America | 68.9-69.6 | 100 | serum | miR-21-5p(up) | RT‒qPCR | 8 | 54 |
|  |  |  | 68.9-69.6 | 100 | serum | miR-125b-5p(down) | RT‒qPCR | 7 | 48 |
| Yang ^[24]^ | 2013 | China | 49.9 | 100 | bone | miR-21-5p(down) | RT‒qPCR | 5 | 5 |
| Zhao ^[59]^ | 2021 | China | UN | 100 | serum | miR-483-5p(up) | RT‒qPCR | 36 | 36 |
| Wang ^[48]^ | 2018 | China | UN | UN | serum | miR-125b-5p(up) | RT‒qPCR | 45 | 15 |
