# Supplementary material for: The characteristic expression of circulating MicroRNAs in osteoporosis: a systematic review and meta-analysis
Source: Front Endocrinol (Lausanne). 2024 Dec 16;15:1481649. doi: 10.3389/fendo.2024.1481649 (PMC11682891; doi:10.3389/fendo.2024.1481649)
Supplement: Supplementary file 1 [file DataSheet1.zip › supplementary files/Supplemental Table 2. The Begg’s and Egger’s Test in STATA of each miRNA..docx]

**Supplemental Table 2.** The Begg’s and Egger’s Test in STATA of each miRNA.

| miRNA | P (Begg’s test) | P (Egger’s test) |
| --- | --- | --- |
| miR-21-5p | 0.084 | 0.929 |
| miR-125b-5p | 0.096 | 0.573 |
| miR-483-5p | 0.278 | 0.497 |
| miR-133a | 0.54 | 1 |
| miR-148a-3p | 0.456 | 0.174 |
| miR-497-5p | 0.290 | 0.602 |
| miR-422a | 0.748 | 0.602 |
| miR-214-3p | 0.361 | 0.117 |
| miR-122-5p | 0.530 | 0.602 |
