# Supplementary material for: The characteristic expression of circulating MicroRNAs in osteoporosis: a systematic review and meta-analysis
Source: Front Endocrinol (Lausanne). 2024 Dec 16;15:1481649. doi: 10.3389/fendo.2024.1481649 (PMC11682891; doi:10.3389/fendo.2024.1481649)
Supplement: Supplementary file 1 [file DataSheet1.zip › supplementary files/Supplemental Figure S3. The funnel plots of each miRNA.docx]

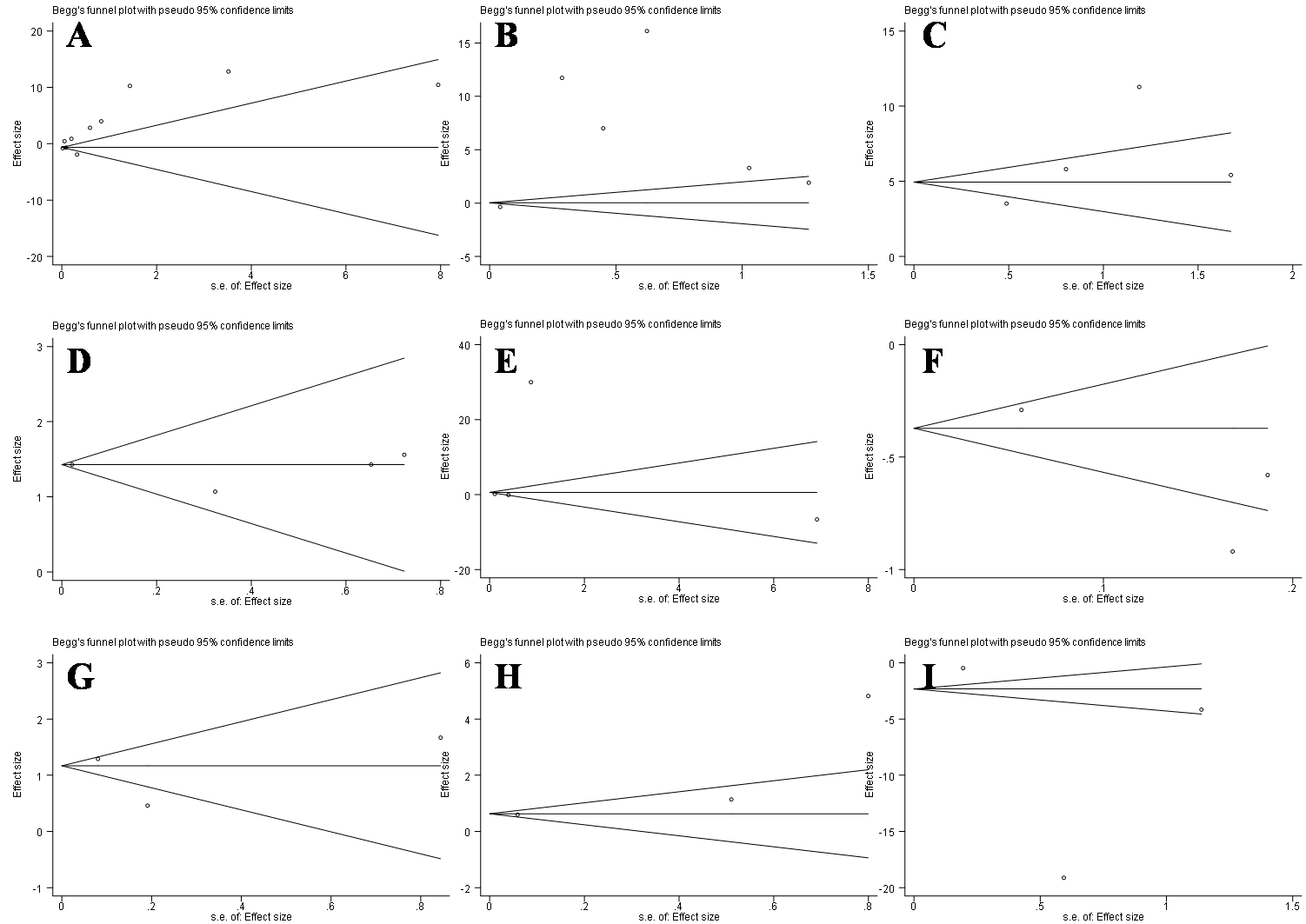


**Supplemental Figure S3.** The funnel plots of each miRNA. A, miR-21-5p; B, miR-125b-5p; C, miR-483-5p; D, miR-133a; E, miR-148a-3p; F, miR-497-5p; G, miR-422a; H, miR-214-3p; I, miR-122-5p
