# Supplementary figures and images for: The characteristic expression of circulating MicroRNAs in osteoporosis: a systematic review and meta-analysis
Source: Front Endocrinol (Lausanne). 2024 Dec 16;15:1481649. doi: 10.3389/fendo.2024.1481649 (PMC11682891; doi:10.3389/fendo.2024.1481649)

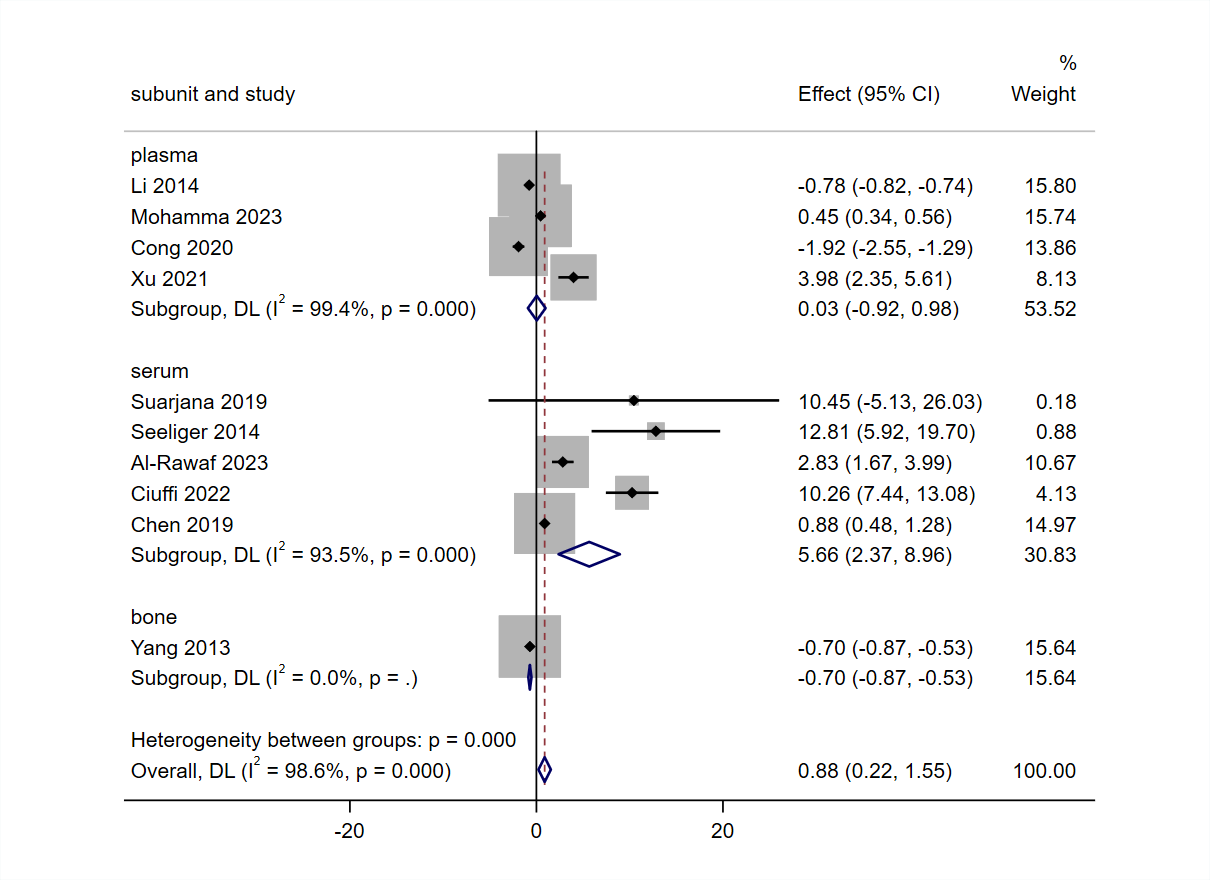


**Supplemental Figure S1.** Subgroup analysis of miR-21-5p.

Supplement: Supplementary file 1 [file DataSheet1.zip › supplementary files/Supplemental Figure S1. Subgroup analysis of miR-21-5p.docx]
